# Supplementary material for: The causal relationship between the human gut microbiota and pyogenic arthritis: a Mendelian randomization study
Source: Front Cell Infect Microbiol. 2024 Nov 26;14:1452480. doi: 10.3389/fcimb.2024.1452480 (PMC11629706; doi:10.3389/fcimb.2024.1452480)
Supplement: Supplementary file 1 [file DataSheet1.zip › mendelian test/ebi-a-GCST90027534.csv_forest.pdf]

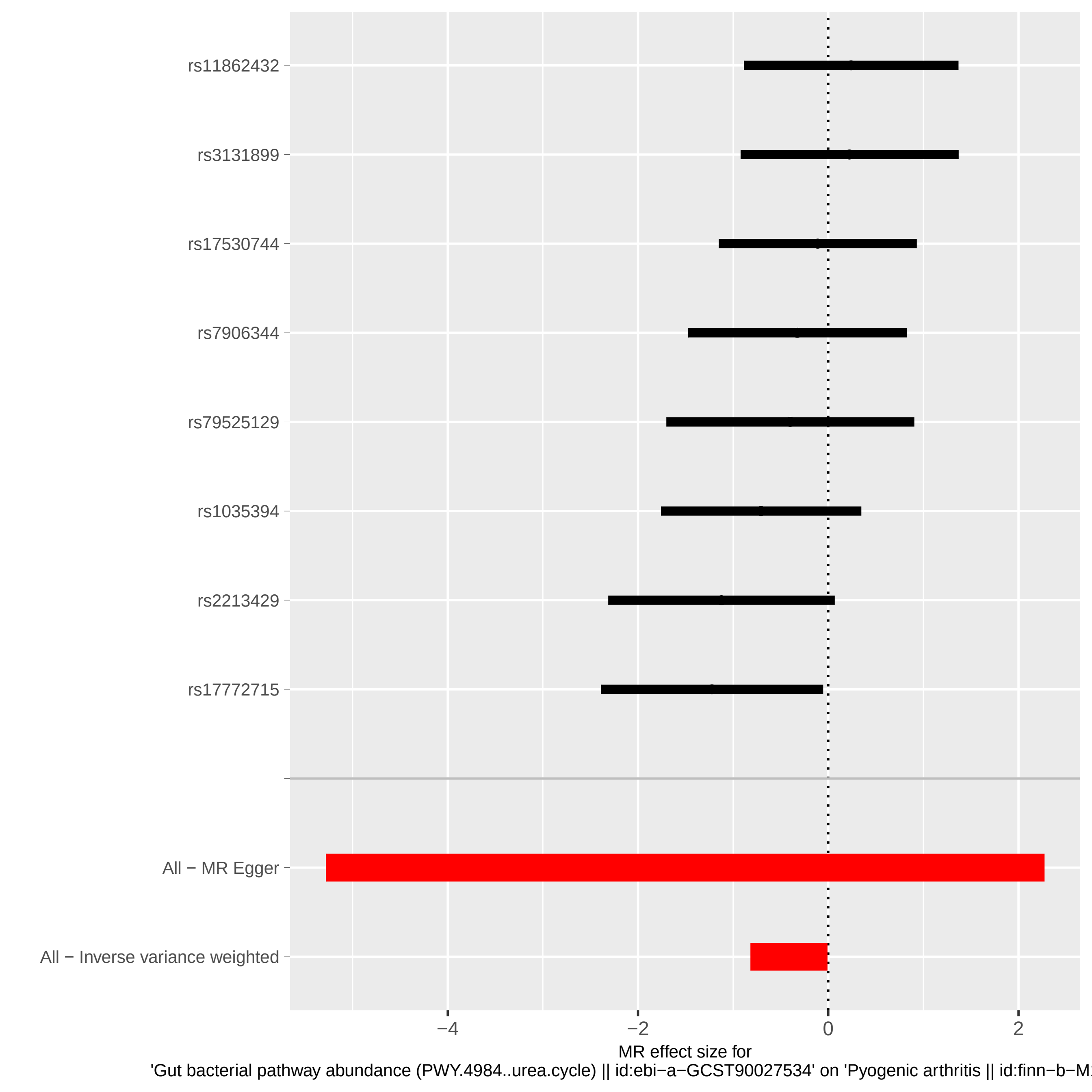

rs11862432
rs3131899
rs17530744
rs7906344
rs79525129
rs1035394
rs2213429
rs17772715
All − MR Egger
All − Inverse variance weighted
−4
−2
0
2
MR effect size for
'Gut bacterial pathway abundance (PWY.4984..urea.cycle) || id:ebi−a−GCST90027534' on 'Pyogenic arthritis || id:finn−b−M
